# TAVR in Cancer Patients: Comprehensive Review, Meta-Analysis, and Meta-Regression

**DOI:** 10.3389/fcvm.2021.641268

**Published:** 2021-08-04

**Authors:** Konstantinos Marmagkiolis, Dominique J. Monlezun, Mehmet Cilingiroglu, Cindy Grines, Joerg Herrmann, Konstantinos Pavlos Toutouzas, Ismail Ates, Cezar Iliescu

**Affiliations:** ^1^University of Texas MD Anderson Cancer Center, Houston, TX, United States; ^2^Department of Medicine, Wayne State University, Detroit, MI, United States; ^3^Mayo Clinic, Rochester, MN, United States; ^4^Department of Cardiology, National and Kapodistrian University of Athens, Athens, Greece; ^5^Department of Cardiology, Bahçeşehir University, Istanbul, Turkey

**Keywords:** TAVR, cancer, cardio-oncology, meta-analysis, aortic stenosis

## Abstract

**Objectives:** This study sought to systematically analyze the available clinical evidence on TAVR therapy in cancer patients with symptomatic severe AS.

**Background:** Aortic stenosis is the most common valvular heart disease in the world. TAVR has expanded the treatment options for this lethal disease process. The safety and efficacy of TAVR in cancer patients has not yet been reliably established. We thus conducted the largest known multi-center meta-analysis on TAVR and cancer status.

**Methods:** We performed a literature search using PubMed, EMBASE, and Cochrane Central Register of Controlled Trials from January 2015 to 2020. Studies that compared the use of TAVR in patients with severe symptomatic aortic stenosis and cancer against patients without cancer were included. Meta-regression was also conducted to determine if common clinical factors modified the possible association between cancer status and TAVR mortality.

**Results:** Five studies with 11,129 patients in the cancer group and 41,706 patients in the control group met inclusion criteria. The short-term mortality in the cancer group was 2.4% compared with 3.3% in the control group (odds ratio: 0.72, 95% confidence interval: 0.63–0.82; *p* < 0.0001). The frequency of stroke was 2.4% compared with 2.7% (odds ratio of 0.87, 95% confidence interval: 0.76–0.99; *p* < 0.04). The frequency of AKI was 14.2% in cancer patients vs. 16.4% (odds ratio of 0.81, 95% confidence interval: 0.76–0.85; *p* < 0.04). The rates of bleeding and need for new pacemaker implantation were not significantly different. Meta-regression demonstrated there was no significant association modifying.

**Conclusions:** On the basis of the results of this meta-analysis TAVR may be a safe and effective therapeutic option for patients with cancer and symptomatic severe aortic stenosis. Larger, longer, and randomized trials are required to adequately test this above hypothesis.

## Introduction

Aortic stenosis (AS) is the most common valvular heart disease in the world with ~500,000 patients with severe aortic stenosis in the United States alone (1). Symptomatic severe AS is associated with dismal prognosis and an average survival of <3 years if left untreated (2). Initial retrospective data suggested that cancer patients with severe AS who underwent surgical aortic valve replacement (SAVR) experienced improved survival, regardless of cancer status (3). As one of the most important advancements of the past 10+ years, Transcatheter Aortic Valve Replacement (TAVR) has expanded the treatment options for this lethal disease process and it is now FDA-approved for patients with inoperable, high, intermediate, and low risk for surgical aortic valve replacement (SAVR) (4). Patients with cancer often carry a high burden of comorbidity and may be deemed to be ideal candidates for TAVR. However, these patients have been traditionally excluded from TAVR randomized controlled trials (RCT); thus the safety and efficacy of TAVR in cancer patients has not yet been reliably established. Indeed, while conditions associated with cancer and cancer therapy (anemia, thrombocytopenia, bleeding diathesis, thrombophilia, and increased frailty) may argue for a transcatheter approach, they may, at the same time complicate transcatheter interventions. This comprehensive review and meta-analysis seeks to systematically analyze the available clinical evidence on TAVR therapy in cancer patients with symptomatic severe AS.

## Methods

A protocol was prospectively developed detailing the specific objectives, criteria for study selection, approach to assess study quality, outcome and statistical methods. We performed a literature search using Pubmed, EMBASE, Cochrane Central Register of Controlled Trials, and Internet-based sources of Information on clinical trials (clinicaltrials.gov) from January 2015 to January 2020. The Medical Subject Heading (MeSH) terms “transcatheter aortic valve implantation” or “transcatheter aortic valve replacement” combined with “cancer,” “malignancy,” or “oncology” were used. No language restrictions were applied. Bibliographies of relevant studies and the “Related Articles” link in PubMed were used to identify additional studies. Published abstracts from the annual meetings of the American College of Cardiology, American Heart Association, European Society of Cardiology, Trans Catheter Therapeutics, Society of Coronary Angiography and Intervention, and Euro Percutaneous Coronary Revascularization, were also identified. Studies comparing the use of TAVR in patients with cancer and patients without cancer were included in the meta-analysis. The study received the proper ethical oversight.

### Data Extraction

Two investigators (K.M. and C.I.) independently reviewed the studies and reported the results in a structured dataset. Studies were evaluated carefully for duplicate or overlapping data. Disparities between investigators regarding the inclusion of each trial were resolved by consensus by a third independent investigator (M.C.). Eligible trials to be included in our meta-analysis had to meet the following criteria: Studies that compared the use of TAVR in patients with severe symptomatic aortic stenosis and either active malignancy or history of cancer vs. patients without cancer. Prespecified data elements were extracted from each trial as follows: sample size, sex, age, history of coronary artery disease (CAD), hypertension, dyslipidemia, diabetes, atrial fibrillation, stroke or transient ischemic attack (TIA), chronic kidney disease (CKD), Euroscore and STS (Society of Thoracic Surgeons) score. The primary endpoints were short-term mortality, post-operative stroke, acute kidney injury (AKI), bleeding and need for pacemaker implantation.

### Statistical Analysis

We used odds ratios (ORs) with 95% confidence intervals (CIs) as the metric of choice for all outcomes. Categorical variables were reported as percentages, and continuous variables as mean SD. Weighted means were used for the pooled estimates of continuous variables. The pooled OR was calculated with the DerSimonian–Lairdmethod for random effects. To assess heterogeneity across studies, we used the Cochran Q *via* a Mantel-Haenszel test based on the pooled OR. Based on the *I*^2^ statistic, values of 25, 50, and 75% were considered as yielding low, moderate, and high heterogeneity, respectively (5–7). Results were considered statistically significant at *p* < 0.05. A funnel plot and the adjusted rank correlation test were used to assess for publication bias with respect to the primary outcome of interest. With the use of a funnel plot, the OR was plotted on a logarithmic scale against its corresponding standard for each study. In the absence of publication bias, one would expect studies of all sizes to be scattered equally right and left of the line showing the pooled estimate of natural log RR. Statistical analyses were performed with RevMan software version 5.3.5 (Cochrane's Informatics and Knowledge Management Department). Meta-regression analyses investigated the effects of study-level characteristics with sex, diabetes, hypertension, dyslipidemia, coronary artery disease, cerebrovascular disease, smoking, chronic kidney disease, atrial fibrillation, and major bleeding represented as proportions with age, European System for Cardiac Operative Risk Evaluation (EuroSCORE), and Society of Thoracic Surgeons (STS) scores represented in their respective standard continuous units. We used the baseline patient traits from the individual studies as independent variables in linear meta-regression on the log-transformed RR of cancer vs. non-cancer on mortality to calculate the variables' meta-regression coefficients with 95% CIs, thus testing if any of the variables were modulators of the effect of cancer vs. non-cancer on mortality. Chemotherapy and anticoagulation were not analyzed given the absence of this data from the respective studies. Meta-regression analysis was performed using Stata version 14.2 (StataCorp LP, College Station, Texas).

## Results

**Figure 1:**
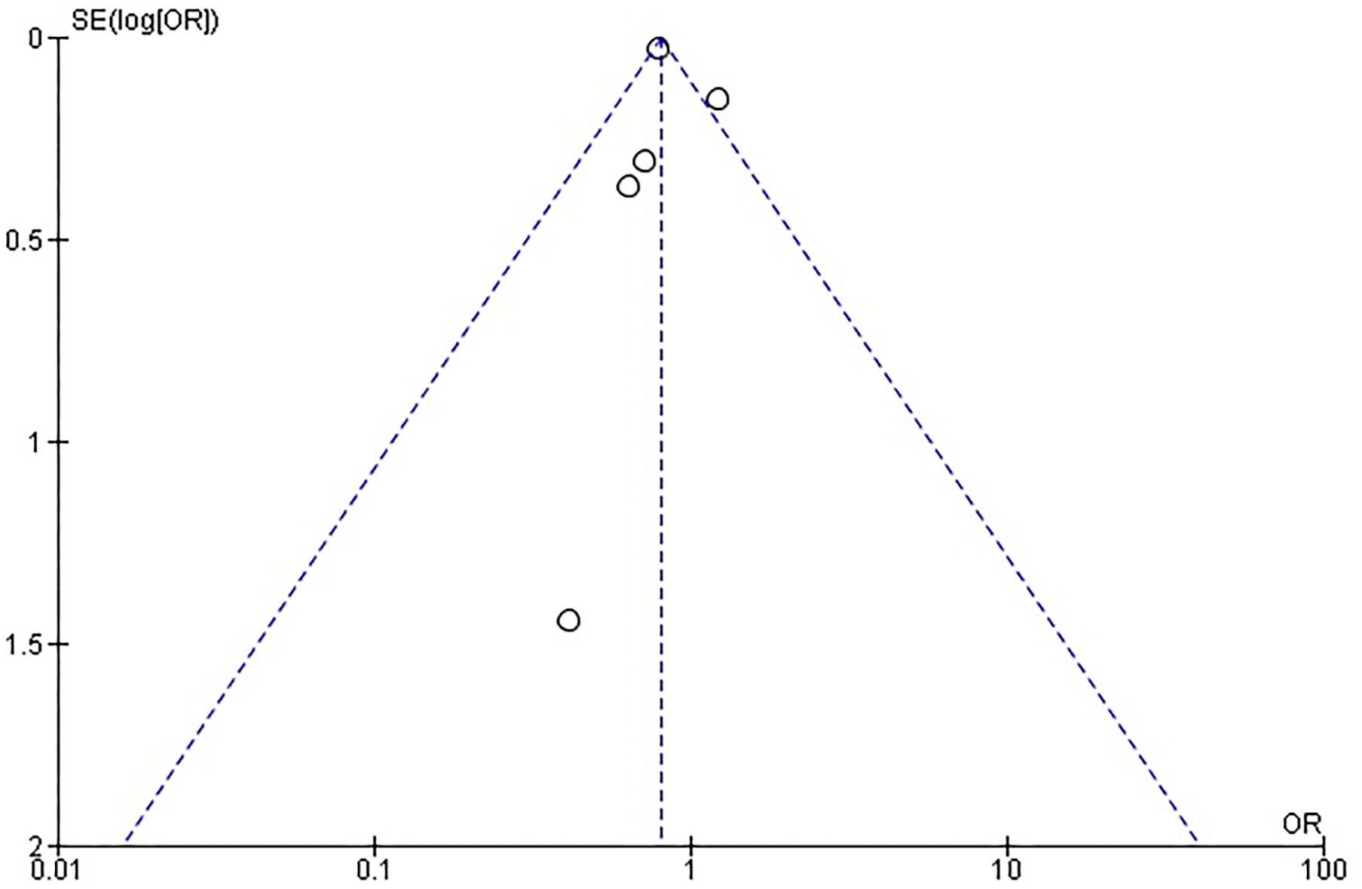
Meta-analysis flow diagram.

**Figure 2:**
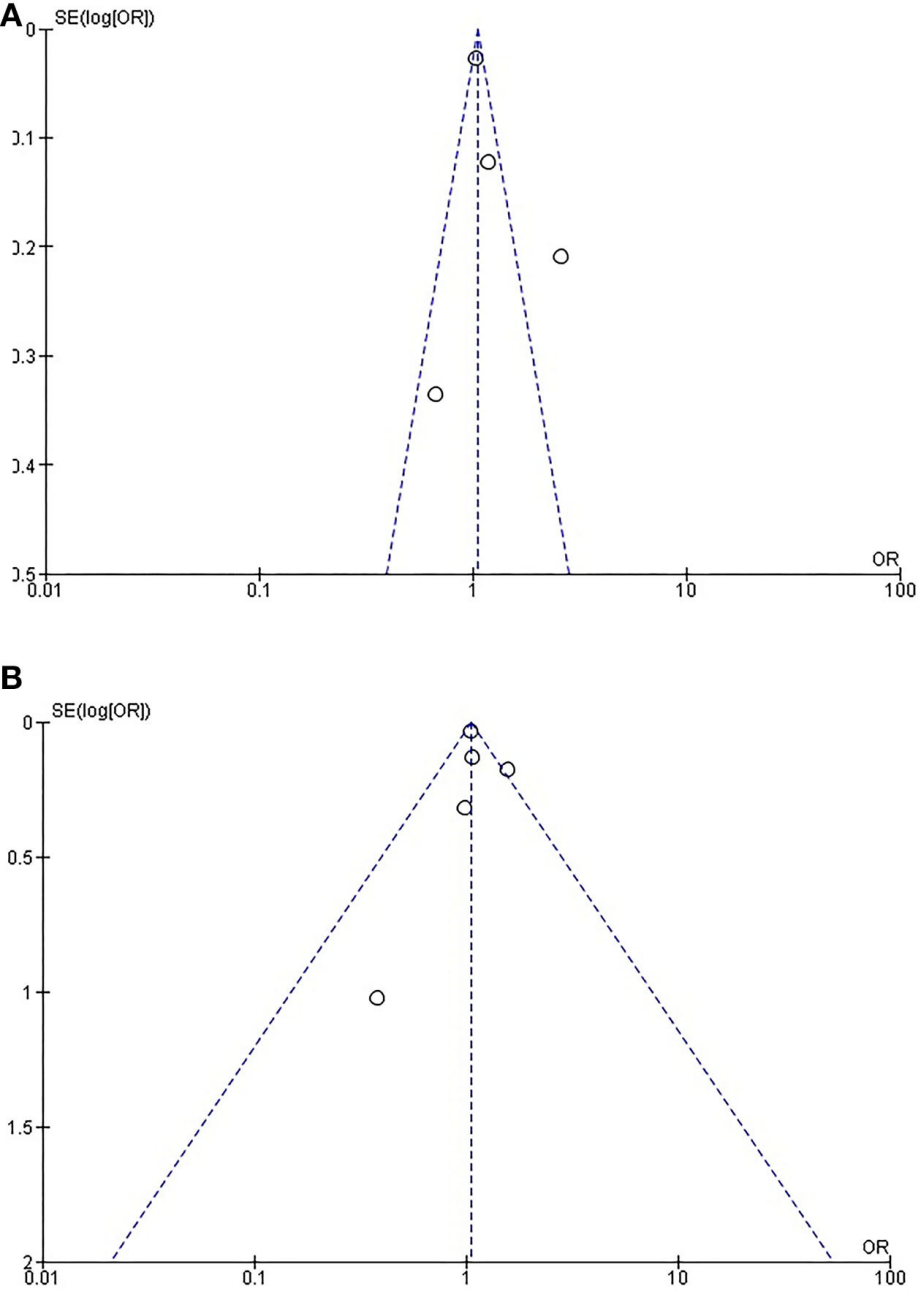
**(A)** Forest plot meta-analysis. **(B)** Forest plot meta-analysis.

Of the 493 citations found, five studies were identified (8–12). Characteristics of the five studies are summarized in Table 1 and Figure 1. All studies were observational. The study from Landes et al. was derived from an international registry while the rest were based on national registries from the United States, Germany, Japan, and Israel. Landes and Watanabe included patients with active cancer while Guha, Berkovitch, and Mangner included patients with active or history of malignancy. Berkovitch and Mangner excluded cancer patients with expected survival <1 year, while Watanabe excluded patients with bicuspid or non-calcified aortic valves, severe aortic insufficiency, and patients dependent on dialysis. All studies utilized balloon expandable and self-expanding except the one from Watanabe which included only balloon expandable valves. The baseline age was comparable in all studies (Table 2). The sample of cancer patients who received TAVR included more male patients with higher rates of underlying CAD and dyslipidemia. The control group included patients with higher rates of diabetes, CKD and atrial fibrillation. Patients with cancer had lower mean EuroScore (4.1 vs. 5.1) and STS scores (4.9 vs. 6.3). The clinical outcomes of the included studies are summarized in Table 3 and Figures 2A,B.

**Table 1:** Characteristics of included studies.

**Study**	**Studied period**	**Location**	**Sample size**	**Cancer definition**	**Exclusion**	**Valve types**
Guha et al. (10)	2012–2015	USA	47,295	Any history of malignancy	None	Both
		NIS National registry				
Landes et al. (8)	2008–2016	International 18 TAVR centers	8,497	Active malignancy	None	Both
Berkovitch et al. (9)	2008–2015	Israel	477	Any history of malignancy	<1 year expectancy	Both
		Single Center				
Mangner et al. (11)	2006–2014	Germany	1,821	Any history of malignancy	<1 year expectancy	74.5% Balloon-expandable
		Single center				
Watanabe et al. (12)	2013–2015	Japan	749	Active malignancy stage >T2 or any malignancy refractory, relapsing, or recurrent	Bicuspid or noncalcified AV, severe AR, or HD dependence	Self-expanding only
		Multi-center registry				
		8 TAVR centers				

**Table 2:** Baseline characteristics of included studies.

	**Study**	**Guha et al. (11)**	**Landes et al. (8)**	**Berkovitch et al. (9)**	**Mangner et al. (11)**	**Watanabe et al. (12)**
Number of patients	Cancer	10,670	222	91	99	47
	Control	36,625	2,522	386	1,471	702
Male,%	Cancer	57.2%	62.0%	52.0%	42.7%	45.0%
	Control	52.6%	45.0%	52.0%	42.2%	33.0%
Age, years	Cancer	81.1	78.8	79.4	80.5	83
	Control	80.8	81.3	81.8	81	85
CAD, %	Cancer	67.8%	35.0%	47.0%	51.8%	26.0%
	Control	68.8%	17.0%	48.0%	53.1%	24.0%
CVA, %	Cancer	14.0%	11.0%	18.0%	10.8%	11.0%
	Control	13.3%	18.0%	14.0%	9.8%	14.0%
DM2, %	Cancer	38.0%	28.0%	34.0%	39.5%	30.0%
	Control	41.5%	36.0%	40.0%	43.6%	25.0%
HTN, %	Cancer	83.5%	76.0%	82.0%	93.5%	75.0%
	Control	83.8%	92.0%	85.0%	93.6%	75.6%
DLP, %	Cancer	68.8%	57.0%	60.0%	N/A	43.0%
	Control	65.7%	87.0%	75.0%	N/A	43.0%
CKD, %	Cancer	36.9%	N/A	24.0%	30.0%	N/A
	Control	37.9%	N/A	22.0%	34.3%	N/A
Afib, %	Cancer	41.4%	N/A	N/A	40.8%	17.0%
	Control	43.4%	N/A	N/A	44.9%	19.0%
Mean EuroScore	Cancer	N/A	4.2	4.5	N/A	3.10
	Control	N/A	5.4	5.4	N/A	3.9
Mean STS score	Cancer	N/A	4.9	4.6	N/A	5.4
	Control	N/A	6.2	5.7	N/A	7.0

*CAD, Coronary artery disease; CVA, Cerebrovascular accident; HTN, Hypertension; DLP, Dyslipidemia; CKD, Chronic kidney disease; Afib, Atrial fibrillation; STS Score, Society of Thoracic Surgeons Score*.

**Table 3:** Clinical outcomes of included studies.

	**Short-term mortality**		**Stoke**		**Acute kidney injury**		**Bleeding**		**Need for pacemaker**	
**Study**	**Cancer**	**Control**	**Cancer**	**Control**	**Cancer**	**Control**	**Cancer**	**Control**	**Cancer**	**Control**
Guha et al. (10)	2.3	3.2	2.4	2.7	14.3	17.3	19.8	19.2	11.3	10.9
Landes et al. (8)	1.8	3.2	0.9	0.9	3.6	5.5	14.4	6.1	19.4	13.7
Berkovitch et al. (9)	1.1	5.2	2.2	3.4	16.5	21.5	N/A	N/A	15.4	15.6
Mangner et al. (11)	6.0	7.6	4.7	4.7	19.3	16.5	41.9	37.9	29.7	28.4
Watanabe et al. (12)	4.3	2.7	2.1	5.6	0.00	2.4	27.7	36.5	2.1	5.4

*Values in %*.

### Short-Term Mortality

Rates of short-term mortality were reported in all trials (Figure 2A). In the study from Guha and Berkovitch, short-term mortality was described as in-hospital deaths. In the rest of the studies, short term mortality was assessed at 30 days. The overall mortality in the cancer group was 2.4% (273 of 11,371) compared with 3.3% (1,391 of 41,706) in the control group. Patients in the cancer group had an odds ratio of 0.72 (95% confidence interval: 0.63 to 0.82; *p* < 0.0001) for short-term mortality compared to the patients without cancer. There was no evidence of statistical heterogeneity among studies (*I*^2^ = 0%; heterogeneity *p* = 0.5). There was no evidence of publication bias for the primary endpoint on visual estimation of the funnel plot (Supplementary Figure 1).

### Periprocedural Acute Cerebrovascular Event or Transient Ischemic Attack

Rates of CVA/TIA were reported in all trials. Only the study from Guha reported both CVA and TIA, while the rest of the studies described only the rates of CVA. The overall stroke rate was 2.4% (264 of 11,126) compared with 2.7% (1,141 of 41,661) in the control group. Patients in the cancer group had an odds ratio of 0.87 (95% confidence interval: 0.76–0.99; *p* < 0.04) for periprocedural stroke compared to the patients without cancer. There was no evidence of statistical heterogeneity among studies (*I*^2^ = 0%; heterogeneity *p* = 0.92). There was no evidence of publication bias on visual estimation of the funnel plot (Supplementary Figure 2).

### Acute Kidney Injury

Rates of AKI were reported in all trials. The overall AKI rate was 14.2% (1,614 of 11,372) in cancer patients compared with 16.4% (6,815 of 41,668) in the control group. Patients in the cancer group had an odds ratio of 0.81 (95% confidence interval: 0.76–0.85; *p* < 0.04) for AKI compared to the patients without cancer. There was significant heterogeneity among studies (*I*^2^ = 49%; heterogeneity *p* = 0.10). There was evidence of publication bias on visual estimation of the funnel plot (Supplementary Figure 3).

### Bleeding

Rates of short-term bleeding were reported in four trials. Guha et al. only reported bleeding necessitating transfusion. The studies from Landes and Watanabe reported any bleeding, while Manger reported all VARC-II bleeding events. The overall bleeding rate was 20.3% (2,298 of 11,280) in cancer patients compared with 19.3% (7,995 of 41,273) in the control group. Patients in the cancer group had an odds ratio of 1.05 (95% confidence interval: 1.00–1.11; *p* < 0.06) for bleeding compared to the patients without cancer. There was significant heterogeneity among studies (*I*^2^ = 86%; heterogeneity *p* < 0.0001), probably due to heterogeneity in the definition of bleeding. There was evidence of publication bias on visual estimation of the funnel plot (Supplementary Figure 4).

### Need for Pacemaker Implantation

Rates of new pacemaker implantation were reported in all trials. The overall rate of new pacemaker implantation was 12.0% (1,372 of 11,380) in the cancer group compared with 11.6% (4,842 of 41,705) in the control group. Patients in the cancer group had an odds ratio of 1.06 (95% confidence interval: 0.99–1.13; *p* < 0.09) for a new pacemaker implantation need compared to the patients without cancer. There was significant heterogeneity among studies (*I*^2^ = 31%; heterogeneity *p* < 0.22). There was evidence of publication bias on visual estimation of the funnel plot (Supplementary Figure 5).

### Meta-Regression

The effects of meta-regression coefficients on mortality were not statistically significant for sex, diabetes, hypertension, dyslipidemia, coronary artery disease, cerebrovascular disease, smoking, chronic kidney disease, atrial fibrillation, major bleeding, age, EuroSCORE, nor STS scores (all *p* > 0.05).

## Discussion

Cardiovascular disease and cancer are the two leading causes of death in developed countries. Despite the increasing prevalence in both, death rates have been steadily declining with the introduction of technology and novel therapies. Tailoring the most optimal and appropriate management for patients with this double jeopardy can be challenging (13). Most cardiovascular conditions in cancer patients can be now safely assessed and managed in the cardiac catheterization lab (14).

Previously, the clinical dilemma to continue cancer treatment in patients with severe AS vs. delaying cancer treatment and undergo surgical aortic valve replacement (SAVR) often favored the former. However, the few patients that underwent SAVR had dramatically better survival, predominantly from improved resilience to anemia, infections/sepsis, and rapid volume changes from chemotherapeutic regimens or hypotension/volume loss during surgical procedures, not uncommon during the cancer treatment roller-coaster (3). The increased access of cancer patients to TAVR dramatically changed clinical decisions minimizing the delays in cancer care from ~2 months to 2 weeks (15). Today most cancer patients undergo AVR before cancer treatment, with the large majority receiving TAVR vs. SAVR. Our meta-analysis tries to answer the next question: what is the procedural and short-term risks of TAVR which may translate to delays in cancer treatment and modified overall survival.

This meta-analysis demonstrates a favorable post-TAVR short-term mortality and remarkable safety. In fact, we observed improved stroke and AKI rates without increased bleeding and need for new pacemaker implantation in cancer patients compared to controls. The convergence of five registries, even after taking into consideration their observational nature, leads to the assertion that there is no longer equipoise but an argument for the application of TAVR in cancer patients. Those results are in contrast to a recent meta-analysis from Bendary et al. (5) which reported higher rates of postprocedural pacemaker, probably due to the non-inclusion of the most recent NIS data from Guha et al.

Valvular disease has been long acknowledged as a serious adverse effect of cancer therapy including radiation and chemotherapy (6, 16). It can potentially occur in >75% of patients who have received RTX (7). Cancer patients are often turned down for surgical AVR due to assumed limited life expectancy or increased risk of bleeding, liver or kidney dysfunction, cognitive dysfunction, scarring from chest radiation or prior open heart surgery (17). Moreover, previous chest radiation therapy results in slower sternal wound healing, aortic root calcification and increased bleeding. Euroscore and STS scores do not take into consideration all the aforementioned factors. However, studies in cancer patients undergoing TAVR after chest radiation do indicate a lower than expected mortality (18). While the current guidelines do not recommend TAVR in patients with life expectancy is <1 year, many cancer survivors do not meet this timeline and even those on active therapy are experiencing continuously improving survival (18–20). There therefore will be a rising need to revisit the option and benefit of TAVR in cancer patients.

Our results are in contrast with the recent metanalysis of Bendary et al., where no difference in short-term mortality was recorded (5). Moreover, these favorable outcomes may not translate to longer term follow up. Indeed, Bendary et al. reported higher 1-year mortality rate in the cancer group, mainly driven by patients in advanced cancer stage. Compared to previous meta-analysis, the addition of the largest study to date from Guha et al., with over 35,000 patients accounts for the significantly different results. In the present study, this short term “cancer paradox” could be partially explained by the lower Euroscores and STS scores in the cancer group. In addition, no differentiation was made between active vs. prior cancer. The favorable outcome on acute kidney injury rates could partially be explained by the lower rates of diabetes and chronic kidney disease in cancer group. There was no difference in the rate of permanent pacemaker implantation between patients with cancer and without cancer. Those results are in contrast to the recent meta-analysis from Bendary et al. which reported higher rates of postprocedural pacemaker, probably due to the lack of inclusion of the most recent NIS data from Guha et al. Interestingly, in the present meta-analysis the presence of cancer was not associated with higher bleeding complications during TAVR. In most centers, today oncologists oversee cancer therapy during TAVR and they often need to temporarily modify cancer therapy or transfuse platelets or other blood products when necessary. Bleeding events is often the most serious concern for cancer patients with severe aortic stenosis referred for surgical aortic valve replacement. Indeed, sternotomy and cardiopulmonary bypass pose an increased risk for bleeding complications in cancer patients. Thus, TAVR represents a viable alternative for those patients.

### Limitations

To date there are no randomized controlled trials on the safety and value of TAVR in cancer patients. As with any meta-analysis, the conclusions drawn from such data are subject to the limitations of the original studies. Patient-level data were not available, precluding subgroup analysis. Our meta-analysis is based on observational studies with all associated inherent bias. Without proper randomization, important selection bias exists for cancer patients who received TAVR after decision from regional multi-disciplinary structural teams. This is reflected in the unequal Euroscore and STS scores between the two groups. The increased rates of diabetes, chronic kidney disease, and atrial fibrillation in the control group may partially explain the unfavorable clinical outcomes compared to the cancer group. We only reported short-term outcomes up to 30 days because most cancer patients resume cancer therapy within 2 weeks. It is possible that those favorable outcomes may not translate to intermediate or long-term follow-up. In longer follow-up, it is clinically challenging to assess whether patient outcomes are due to post-operative TAVR complications, cancer therapy or the natural history of the malignancy itself. There was heterogeneity in the description of short-term mortality, stoke or TIA and bleeding rates. Moreover, there was important heterogeneity and publication bias in acute kidney injury, need for pacemaker and bleeding rates. The differences in the definition of bleeding and acute kidney injury may be a possible explanation. The use of different types of TAVR platforms may explain the heterogeneity in the rates of new pacemaker implantation. We did not report procedural outcomes (i.e., rates of transfemoral access, use of more than one valves, conversion to open surgery, coronary obstruction, tamponade, annular rupture, and valve migration) because of the limited data and description in our included manuscripts. Our analysis did not differentiate active cancer with prior cancer history, different types or stages of cancer, type of chemotherapy, radiation therapy or timing of TAVR relative to diagnosis or treatment of cancer. It is probable that those factors may impact clinical outcomes. Our analysis also lacked cost analysis which can vary significantly from cancer vs. non-cancer patients and affect treatment availability and clinical outcomes, and combined with the above lack of clinical long-term endpoints particularly median survival, challenge more confident and comprehensive interpretation of the data and the suitability of cancer vs. non-cancer patients for AS treatments. Thus, it must be stressed that cautious interpretation of these results is required as strictly hypothesis generating only. Yet, the above findings are consistent with a growing body of recent literature including Lind et al. (21) demonstrating in a larger longitudinal cohort study suggesting that cancer vs. non-cancer have similar short-term complications and survival (though with worse long-term survival which is unclear whether this is due to the underlying cancer).

Given the notable selection bias associated with the above factors, we sought to improve the external and internal validity of the results using the more sophisticated meta-regression technique to demonstrate that common factors typically seen in clinical practice and in the literature to modify the relationship of cancer vs. non-cancer on TAVR mortality did not appear to do so at least in the included studies. This gives some degree of greater confidence that the main findings in this study (that includes a large meta-analysis level of patients with advanced meta-regression techniques) may be genuine hypothesis-generating findings warranting larger, longer, and randomized trials on this topic.

## Conclusion

This meta-analysis demonstrates lower rates of short-term mortality, stroke and acute kidney injury without higher rates of bleeding and pacemaker implantation in cancer patients who undergo TAVR for the management of symptomatic severe AS, compared to patients without cancer. Larger randomized controlled trials are needed to assess the value of TAVR in different types and stages of cancer and to identify the subgroups with the most benefit.

## Data Availability Statement

Publicly available datasets were analyzed in this study. This data can be found here: PubMed, EMBASE, and Cochrane Central Register of Controlled Trials.

## Author Contributions

KM, MC, and JH extracted the data. KM and DM performed the analysis. KM, DM, MC, CG, JH, KT, IA, and CI interpreted the results, drafted the manuscript, revised it, and approved its submission. All authors contributed to the article and approved the submitted version.

## Conflict of Interest

The authors declare that the research was conducted in the absence of any commercial or financial relationships that could be construed as a potential conflict of interest.

## Publisher's Note

All claims expressed in this article are solely those of the authors and do not necessarily represent those of their affiliated organizations, or those of the publisher, the editors and the reviewers. Any product that may be evaluated in this article, or claim that may be made by its manufacturer, is not guaranteed or endorsed by the publisher.

## Supplementary Material

Supplementary Figure 1 Funnel plot for short-term mortality.

Supplementary Figure 2 Funnel plot for stroke.

Supplementary Figure 3 Funnel plot for acute kidney injury.

Supplementary Figure 4 Funnel plot for bleeding.

Supplementary Figure 5 Funnel Plot for need for pacemaker.
