# Supplementary figures and images for: TAVR in Cancer Patients: Comprehensive Review, Meta-Analysis, and Meta-Regression
Source: Front Cardiovasc Med. 2021 Aug 4;8:641268. doi: 10.3389/fcvm.2021.641268 (PMC8371265; doi:10.3389/fcvm.2021.641268)

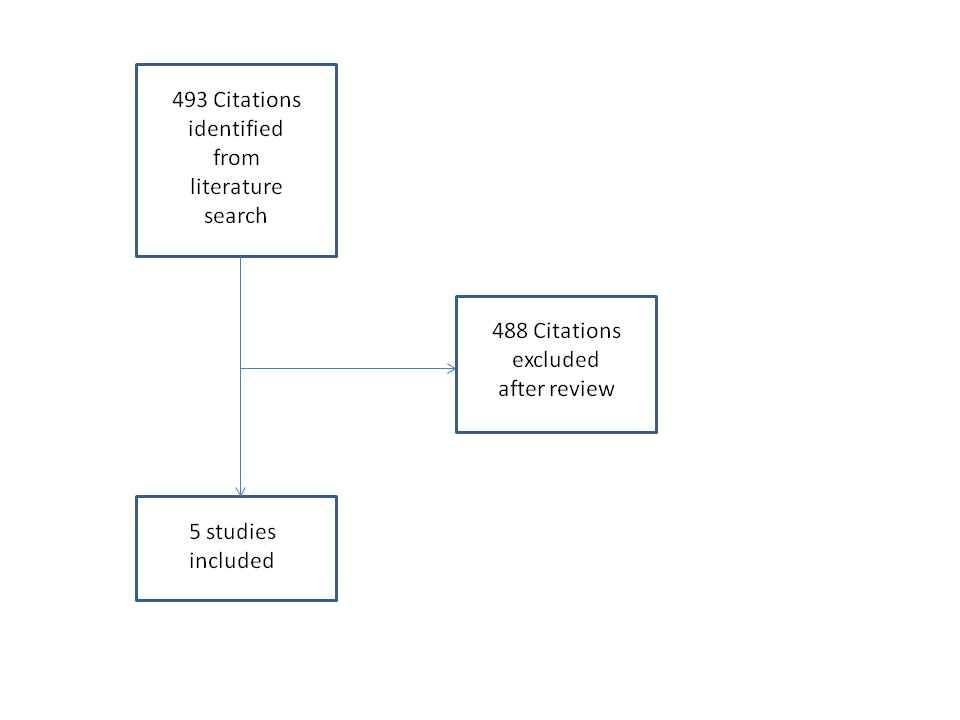

Supplement: Supplementary Figure 1 — Funnel plot for short-term mortality. [file Image_1.TIF]

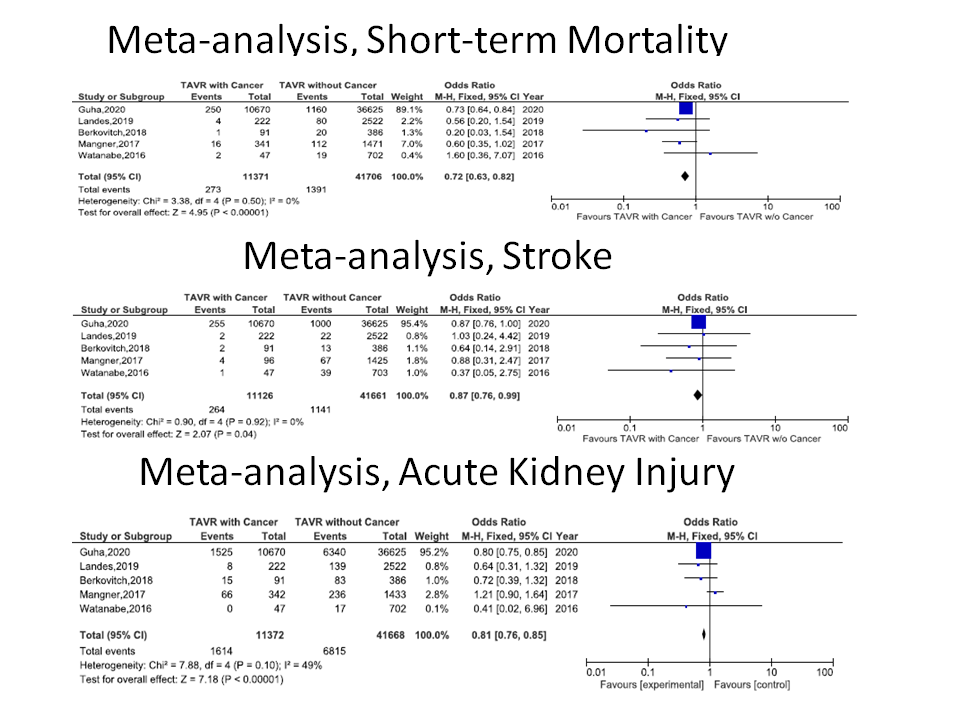

Supplement: Supplementary Figure 2 — Funnel plot for stroke. [file Image_2.TIF]

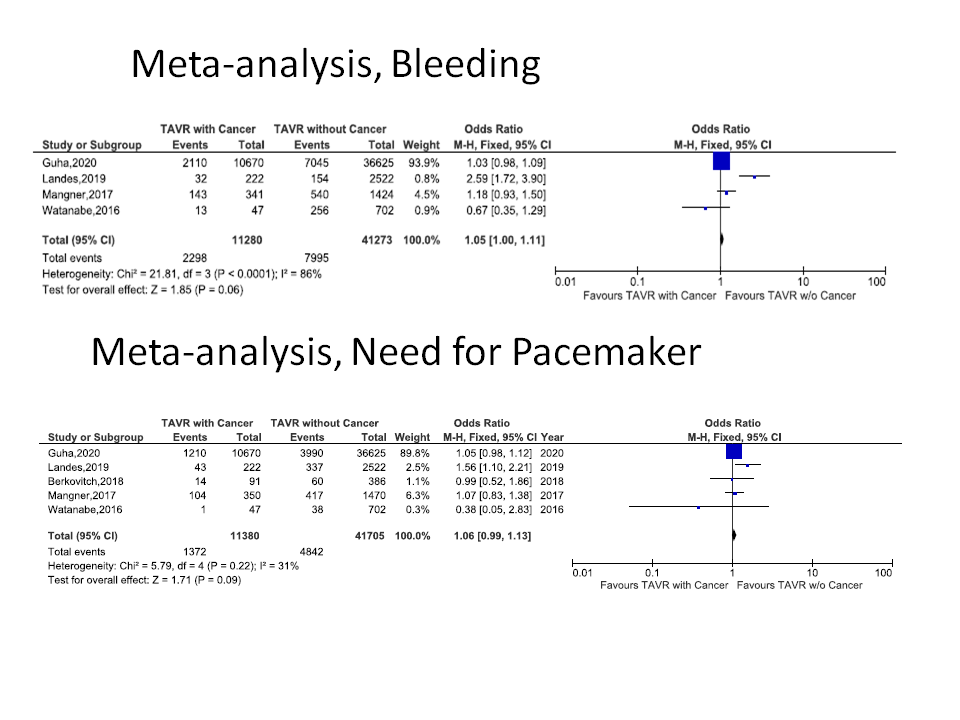

Supplement: Supplementary Figure 3 — Funnel plot for acute kidney injury. [file Image_3.TIF]

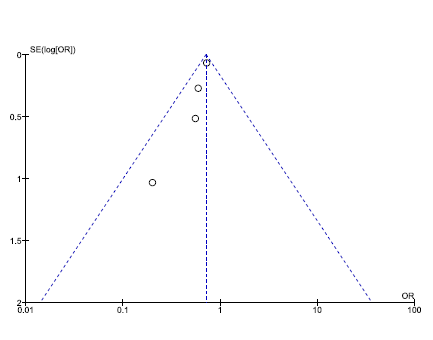

Supplement: Supplementary Figure 4 — Funnel plot for bleeding. [file Image_4.TIF]

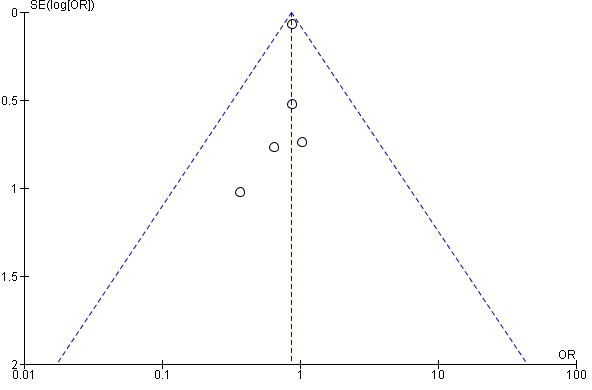

Supplement: Supplementary Figure 5 — Funnel Plot for need for pacemaker. [file Image_5.TIF]
